# A Brief Review of the Application of Neuroergonomics in Skilled Cognition During Expert Sports Performance

**DOI:** 10.3389/fnhum.2019.00278

**Published:** 2019-08-16

**Authors:** Sok Joo Tan, Graham Kerr, John P. Sullivan, Jonathan M. Peake

**Affiliations:** ^1^School of Biomedical Sciences, Institute of Health and Biomedical Innovation, Queensland University of Technology, Brisbane, QLD, Australia; ^2^Sport Performance Innovation and Knowledge Excellence, Queensland Academy of Sport, Brisbane, QLD, Australia; ^3^School of Exercise and Nutrition Sciences, Institute of Health and Biomedical Innovation, Queensland University of Technology, Brisbane, QLD, Australia

**Keywords:** sports, cognitive performance, neuroergonomics, cognition, direct current potential, neuroimaging

## Abstract

The elite sports environment provides a unique setting for studying human performance, where both cognitive and physical demands are high. Successful performance in sport is contingent upon key cognitive skills such as attention, perception, working memory and decision-making. The demands of competitive sport also increase loading on the central nervous system (CNS). Neuroimaging methods such as functional magnetic resonance imaging (fMRI), functional near infrared spectroscopy (fNIRS) and electroencephalography (EEG) offer the potential to investigate the cognitive demands of sport, neuroplasticity of athletes, and biofeedback training. However, practical and technical limitations of these methods have generally limited their use to laboratory-based studies of athletes during simulated sporting tasks. This review article, provides a brief overview of research that has applied neuroimaging technology to study various aspects of cognitive function during sports performance in athletes, alternative methods for measuring CNS loading [e.g., direct current (DC) potential], possible solutions and avenues of focus for future neuroergonomics research in sport.

## Introduction

In many sports, both exceptional physical abilities and cognitive skills such as attention, perception, working memory and decision-making are necessary for success at the highest level. In contrast to our knowledge on the many physiological adaptations of athletic training, the neurocognitive characteristics of peak performance in sports are much less understood. Advances in mobile neuroimaging technology have made it possible to investigate the brain activity of athletes while they execute skilled movements in their normal environment (e.g., on a basketball court). However, the speed of information processing and movement artifacts still pose many challenges for studying the neurological and neurophysiological processes underpinning cognitive skills in elite sports. Due to a lack of suitably robust technologies, investigations involving high levels of cognitive processing important for sports performance have to date been restricted to the laboratory. In this review article, we provide a brief overview of: (1) cognitive functions that are important and can be measured in sport/athletes; (2) current technology available for neuroergonomic research in sport/athletes, including its practical and technical limitations; (3) previous neuroergonomic research in sport/athletes that has used such technology; and (4) issues that are unresolved or unknown in neuroergonomics research in sport/athletes. We also propose some directions for future research.

## Sports-Related Cognitive Processing

Most sports-related research on cognitive processing has compared differences in the activation and structure of brain regions between expert and novice participants to investigate training-induced neuroplasticity (Aglioti et al., 2008; Jin et al., 2011; Abreu et al., 2012; Balser et al., 2014a, b; Makris and Urgesi, 2015; for reviews, see Nakata et al., 2010; Smith, 2016; Perrey and Besson, 2018). Using electroencephalography (EEG), which measures electrical activity of the brain (usually from scalp electrodes), studies have revealed differences between experts and novices. Specifically, differences have been identified in EEG spectral power and lateralization, motor-related cortical potentials, sensory evoked potentials, and event-related potentials (ERPs; Nakata et al., 2010). When spectral power of EEG over the frontal midline was analyzed from recordings during laboratory-based basketball free throws (Chuang et al., 2013) and simulated golf putting (Babiloni et al., 2008), successful performances were associated with greater stability of low-frequency theta waves (4–6 Hz), but smaller amplitude of high-frequency alpha waves (10–12 Hz). In general, laboratory-based studies comparing athletes from various sports (e.g., fencing, gymnastics, karate, kendo and rifle shooting) with non-athletes have attributed smaller motor-related cortical potentials (Kita et al., 2001; Di Russo et al., 2005; Del Percio et al., 2008), shorter latency (Delpont et al., 1991) and smaller peak amplitude (Del Percio et al., 2007) of visual evoked potentials in athletes to greater neural efficiency (i.e., reduced cortical activation). ERPs, in particular, the P300 component, have been widely investigated and found to be associated with cognitive processing. Larger P300 amplitudes observed in baseball players (Nakamoto and Mori, 2008) and fencers (Di Russo et al., 2006) compared with non-athletes represent greater motor inhibitory response during go/no-go tasks. In addition to larger amplitudes, when athletes performed a lower-limb odd-ball task, shorter P300 latencies were observed compared with non-athletes (Iwadate et al., 2005). However, these differences between soccer players and non-athletes were not found when both groups performed an upper-limb task (Iwadate et al., 2005). The authors attributed the findings to neuroplastic changes as a result of extensive training in skill-specific movements. Such results suggest that it may be valuable to develop more robust systems for neuroergonomic assessment during high levels of cognitive and physical performance.

## Cognition in Sports

### Anticipation and Perception

Perhaps one of the most important perceptual-cognitive and perceptual-motor skills in sport is the ability to direct and focus attention on task-relevant visual information. This ability allows athletes to infer the intention and anticipate the action of opponents, or predict the movement of an object. A number of studies have investigated the neurophysiology of action anticipation in expert sport performances (Smith, 2016). These studies identified brain areas that could be implicated in sports-relevant cognitive tasks. Various tasks such as prediction of success in basketball free throws, trajectory of shots in badminton, hockey, tennis and volleyball, and movement of opponents in soccer were examined. However, there were some important limitations to these studies. First, the studies were not performed in situations that included cognitive demands, psychological stress and physical load of actual training or competition. Second, the studies involved responses to stimuli that are not closely related to sports performance (e.g., clicking a mouse or pressing a button). Last, only half the studies included video recordings captured from an athlete’s viewpoint, as opposed to that of a coach or spectator. These three factors reduce the value of these studies for understanding attention, perception and anticipation in sports settings.

A review by Karlinsky et al. (2017) outlined how functional magnetic resonance imaging (fMRI; which measures activity of brain regions by detecting small changes associated with cerebral blood oxygenation and flow) can be used to understand the action observation network and mirror neuron system while athletes execute skills. Evidence from studies outside of sport suggests that this neuronal network or system involve areas of the brain such as the inferior frontal gyrus, superior and inferior parietal lobules, inferior parietal sulcus, and dorsal and ventral premotor cortices (Karlinsky et al., 2017). However, due to the limited availability of portable fMRI equipment, it is currently difficult to image the brain during large dynamic movement with high spatial resolution. On the other hand, the development of other hemodynamic neuroimaging techniques such as functional near-infrared spectroscopy (fNIRS) and transcranial Doppler sonography has created new opportunities for future research on brain activity during sport.

### Attention and Cognitive load

In dynamic team sport environments, when and where athletes direct their attention will influence perception and decision-making. In turn, this will determine behavior and performance outcomes. Maintaining such vigilance and cognitive control is a competitive advantage. Neuroscientific evaluation of attention and cognitive performance has largely supported the multiple resources theory. Briefly, this theory states that resources for cognitive processing are limited and need to be divided between tasks (Wickens, 2007). For example, one study using fNIRS to monitor experienced air traffic controllers demonstrated that the prefrontal cortex was activated when the number of aircraft increased during complex air traffic control tasks (Ayaz et al., 2012). The authors proposed that this increase in prefrontal cortex activity indicates a greater cognitive load. A different air traffic control study found that reliable cueing (i.e., when a prompt was given prior to critical events) helped to maintain cerebral blood flow and performance efficiency. These two variables also correlated with the reliability of cues (Hitchcock et al., 2003). Although there is no direct evidence from athletes in the field—particularly in team sports—the ability to detect and respond only to salient cues may result in more efficient brain metabolism. This is likely to delay the decline any cognitive performance during demanding tasks and increase overall athletic performance.

Athletes often report decreased perceived effort, despite an increase in the cognitive and/or physiological demands of the activity during peak performance. This phenomenon is commonly referred to as flow or being “in the zone.” Flow is facilitated by focus or directed attention (Swann et al., 2012). Recognizing the lack of knowledge about the neurophysiology of flow in sports, Harris et al. (2017) discussed the neurocognitive processes that underly the attentional processes of flow by drawing on the findings from research outside sports. Of note, they identified a study that used fMRI to examine flow states during a 12-min video game. The study reported reduced activity in the rostral anterior cingulate cortex during periods of increased focus (Klasen et al., 2012). This finding suggests that more efficient neural circuits are employed during high levels of focus, and the reduced energy requirement is likely to lead to a decrease in perceived effort. Further research is needed to investigate the neurophysiological basis of flow states during sporting activity.

Some neuroergonomics research indicates additive effects of cognitive and physical loading on brain activity. Using fNIRS, Mehta and Parasuraman (2014) demonstrated that cognitive and physical fatigue induced a greater decrease in blood oxygenation in the prefrontal cortex compared with physical fatigue only. Conversely, using EEG, Xu et al. (2018) observed that cognitive and physical fatigue caused more rapid changes in brain activation, functional connection and complexity compared with mental fatigue.

### Gaze and Visual Search Behavior

To gain further insight into the visual search strategies of skilled performers, eye-tracking has been used to study patterns of eye movements, such as saccades, fixations, and smooth pursuit tracking (Williams et al., 1999). The most commonly used technique to study patterns of eye movements involves using video to evaluate pupil and corneal reflection (Williams et al., 1999; Duchowski, 2017). This technique assumes that the reflection of a light source (usually infrared) is a function of eye position, and thus gaze direction and point of fixation. When the visual search behaviors of expert performers across various sports are compared with non-athletes, experts tend to exhibit fewer fixations of longer durations (Mann et al., 2007). This prolonged fixation is also known as “quiet eye” (QE) period. A meta-analysis on QE and sport performance provides further evidence that QE period is indeed longer in experts compared with novices, and QE training can improve performance (Lebeau et al., 2016).

More recently, mobile eye-tracking devices such as the Pro Glasses 2 manufactured by Tobii have made it possible to record eye movements with neuroimaging methods (e.g., EEG, fNIRS), and synchronize this assessment with other physiological measurements such as galvanic skin response, heart rate and respiration rate. Using this sort of integrated network of sensors can provide important information on how various physiological system are coordinated and could provide valuable opportunity for skill development in sport.

### Neurofeedback and EEG-Based Biofeedback

A high level of self-regulation is necessary to achieve peak cognitive performance. Various biofeedback modalities (notably heart rate variability and surface electromyography) have been used to enhance performance psychology in athletes (Rijken et al., 2016; Jiménez Morgan and Molina Mora, 2017; Rusciano et al., 2017). EEG-based biofeedback (i.e., neurofeedback) has also been shown to improve athletic performance (Vernon, 2005; Cheng et al., 2015; Mirifar et al., 2017; Liu et al., 2018; Xiang et al., 2018). EEG studies on expertise and skilled performance have mostly reported on changes or differences in alpha and beta power, which are often interpreted as reflecting cortical activation and sensorimotor rhythm, respectively. Accordingly, neurofeedback training has generally focused on modulating cortical activation and sensorimotor rhythm.

Due to the technical expertise required in setting up and interpreting EEG recording for neurofeedback training, athletes have not been able to engage independently in such practice. There are currently few qualified professionals working with athletes. In addition, neurodiversity and variability in EEG signals between individuals have generally been overlooked, but are critically important when developing neurofeedback training within elite sport. Using individual (as opposed to population) norms likely improves the safety and effectiveness of neurofeedback training. Fortunately, recently developed consumer wearables use proprietary algorithms to provide individualized feedback. For example, the Canadian-based company InteraXon has developed portable headbands (Muse^TM^ and Muse 2^TM^) that measure EEG from frontal [at Fpz (reference), AF7 and AF8 according to the International 10–20 System] and temporal (TP9 and TP10) electrodes. This portable system has been used successfully to quantify components of ERPs (N200 and P300) during a standard “oddball” task to assess visual attention (Krigolson et al., 2017). This technology has proven effective for recording EEG from large numbers of users over 9 months of biofeedback training (Hashemi et al., 2016). Integrating EEG with other sensors such as accelerometer to monitor head movement and a pulse oximeter to measure heart rate variability provides strong potential for advanced biofeedback training in athletic populations.

### Cognitive Recovery and Assessment of Functional State Using Direct Current Potential

The neurocognitive changes that occur during recovery from exercise are not well understood. However, they are important for understanding and optimizing perceptual-motor adaptations to training and injury rehabilitation (e.g., concussion). This subsection discusses a less commonly used method for assessing the central nervous system (CNS)—direct current (DC) potential.

Recording DC potential can provide an alternative to traditional neuroimaging methods for measuring changes in excitation of the brain during periods of recovery between training and competition. DC potential originating from the brain can be measured both intracranially and non-invasively, relative to a distant part of the body (e.g., base of the thumb). However, because it has a very low frequency (0–0.5 Hz), DC potential is typically filtered out during data processing to remove movement artifacts. DC potential has been recorded and studied since the mid-20th century (Aladzhalova, 1964; Caspers et al., 1980; Ilyukhina, 1982; Ilyukhina et al., 1982; Altenmüller, 1989; Pleydell-Pearce, 1994), but much of the research has not been easily accessible because it was conducted in the former Soviet Union and has not been translated.

In principle, values for DC potential reflect the “readiness” or adaptability of the CNS to respond to cognitive and/or physical loads (Ilyukhina et al., 1982; Ilyukhina, 2013; Heishman et al., 2018). One study used DC potential as an index of CNS readiness, and combined this with measurements of heart rate variability as an index of overall readiness in 10 elite male basketball players. Higher CNS and overall readiness were associated with better countermovement jump height and power (Heishman et al., 2018). The results of this study provide support for using DC potential on its own—and in combination with heart rate variability—as a relatively simple method to monitor changes in performance capacity in athletes.

## Portable Neurophysiological Systems for Sports Research

EEG and fNIRS are the most commonly used and non-invasive methods to record brain electrical and metabolic activities during outdoor walking (De Vos et al., 2014) and cycling (Piper et al., 2014), and treadmill walking and running (Gramann et al., 2010; Gwin et al., 2010; Roeder et al., 2018). Commercially available high-density EEG (e.g., Twente Medical Systems International B. V. and Brain Products GmbH) and fNIRS systems (e.g., NIRx Medical Technologies and Artinis Medical Systems) have made multi-modal measurements possible during everyday human behavior in the field (Perrey and Besson, 2018). However, the use of these systems in most sports training and competition is still uncommon due to their inherent limitations. Factors affecting signal quality such as movement artifacts and sweating (among other methodological concerns) have been discussed in detail in several articles (Pontifex and Hillman, 2008; Gramann et al., 2014; Reis et al., 2014).

Recent fNIRS studies in non-athletes have found that hemodynamic responses in bilateral superior parietal lobes vary as a function of physical load during low- to moderate-intensity barbell squat (Kenville et al., 2017). Another study demonstrated that the shape and amplitude of the deoxyhemoglobin response curve for the motor cortex was not significantly different between indoor and outdoor cycling (albeit on a straight and level bike lane; Piper et al., 2014). In a case-study on table tennis, hemodynamic responses in motor and premotor cortices were different between predictable and unpredictable ball conditions (Balardin et al., 2017). These studies suggest that currently available portable fNIRS systems may offer some useful insights for field-based research in certain sports. Interested readers are referred to a recent publication by Ayaz and Dehais (2019) which provides dedicated methodology chapters on the use of EEG and fNIRS for neuroergonomics and other applications.

## Future Research and Application

Traditional neuroimaging techniques are limited by insufficient spatial (e.g., EEG) and temporal (e.g., fMRI) resolutions. These technical issues have largely restricted previous sports-related investigations to laboratory-based research. Improvements in signal-to-noise ratio and the ability to dissociate EEG signals from potentials of non-brain sources (e.g., skin and muscles) using mobile brain and body imaging systems are necessary before these systems can be used to greater effect in sports settings. Other issues related to prolonged physical exertion (for example, DC drift due to changes in skin temperature and electrochemical properties, and fluctuations in subcutaneous blood flow at electrode sites) also need to be resolved (Pontifex and Hillman, 2008). Some methodological solutions for measuring brain and body dynamics in mobile humans, and relevant hardware and software advancements (e.g., wireless EEG systems, or active electrodes to reduce noise due to movement artifacts) have been discussed elsewhere (Reis et al., 2014). Examples of neuroimaging systems that are more suitable for sports research can also be found in a publication by Perrey and Besson (2018).

**Figure 1:**
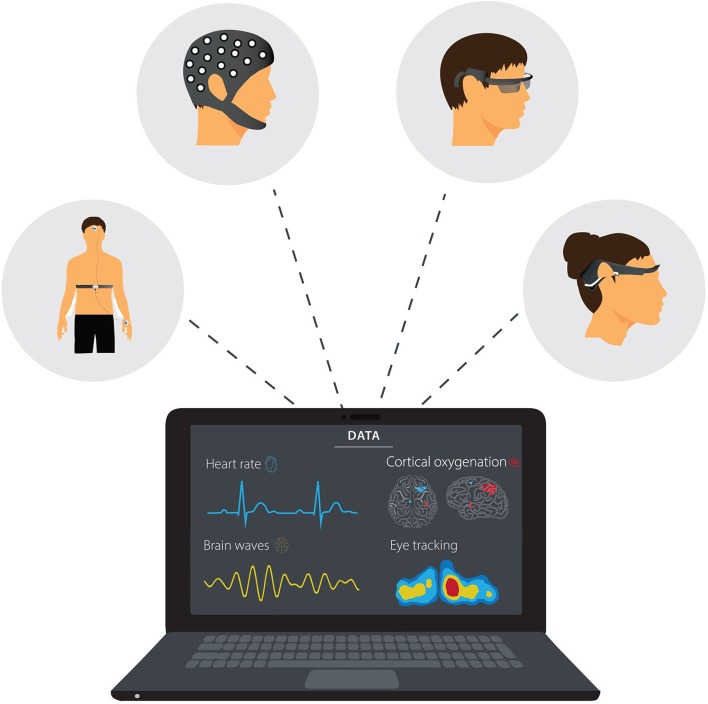
Examples of wearable technology used for neuroimaging and measuring activity of the autonomic nervous system and eye movement in laboratory-based and field studies.

Progress in neuroergonomics research will not only inform the relationship between aspects of cognition (e.g., attention, perception, working memory, decision-making) and sports performance. It will also improve our knowledge about the neurocognitive mechanisms underlying skill development. Longitudinal studies of learning and how previous experiences influence neurophysiological and behavioral responses will provide a better understanding of training-induced neuroplasticity (e.g., the sensitivity of action observation network or mirror neuron system to training). Future research could also investigate possible neuroanatomical indicators of athletic success for different types of sports. Regular neuroergonomic assessments such as mobile EEG, fNIRS and DC potential in the field can also assist in developing neurofeedback training for performance, monitoring recovery and return to sport following injuries like concussion, and long-term functioning and stability/resilience of the athlete. Finally, integrating neurophysiological measurements with other indices of performance (e.g., patterns of eye movement, heart rate, and physical load) using reliable wearable technologies (Figure 1) would allow a deeper understanding of skilled performances in their natural or normal environments.

## Author Contributions

ST drafted, edited and approved the final version of the manuscript. GK, JS and JP edited and approved the final version of the manuscript.

## Conflict of Interest Statement

The authors declare that the research was conducted in the absence of any commercial or financial relationships that could be construed as a potential conflict of interest.
